# Essential oils from two *Allium* species exert effects on cell proliferation and neuroblast differentiation in the mouse dentate gyrus by modulating brain-derived neurotrophic factor and acetylcholinesterase

**DOI:** 10.1186/s12906-016-1384-6

**Published:** 2016-11-03

**Authors:** Hyo Young Jung, Kwon Young Lee, Dae Young Yoo, Jong Whi Kim, Miyoung Yoo, Sanghee Lee, Ki-Yeon Yoo, Yeo Sung Yoon, Jung Hoon Choi, In Koo Hwang

**Affiliations:** 1 Department of Anatomy and Cell Biology, College of Veterinary Medicine, Research Institute for Veterinary Science, Seoul National University, Seoul, 08826 South Korea; 2 Department of Anatomy, College of Veterinary Medicine and Institute of Veterinary Science, Kangwon National University, Chuncheon, 24341 South Korea; 3 Food Analysis Center, Korea Food Research Institute, Sungnam, 13539 South Korea; 4 Department of Oral Anatomy, Research Institute of Oral Sciences, College of Dentistry, Gangneung-Wonju National University, Gangneung, 25457 South Korea

**Keywords:** Acetylcholinesterase, Brain-derived neurotrophic factor, Chive, Garlic, Neurogenesis

## Abstract

**Background:**

In the present study, we investigated the effects of oil products from two *Allium* species: *Allium sativum* (garlic) and *Allium hookeri* (Chinese chives) on cell proliferation and neuroblast differentiation in the mouse dentate gyrus.

**Methods:**

Using corn oil as a vehicle, the essential oil from garlic (10 ml/kg), or Chinese chives (10 ml/kg) was administered orally to 9-week-old mice once a day for 3 weeks. One hour following the last treatment, a novel object recognition test was conducted and the animals were killed 2 h after the test.

**Results:**

In comparison to the vehicle-treated group, garlic essential oil (GO) treatment resulted in significantly increased exploration time and discrimination index during the novel object recognition test, while Chinese chives essential oil (CO) reduced the exploration time and discrimination index in the same test. In addition, the number of Ki67-immunoreactive proliferating cells and doublecortin-immunoreactive neuroblasts significantly increased in the dentate gyrus of GO-treated animals. However, administration of CO significantly decreased cell proliferation and neuroblast differentiation. Administration of GO significantly increased brain-derived neurotrophic factor (BDNF) levels and decreased acetylcholinesterase (AChE) activity in the hippocampal homogenates. In contrast, administration of CO decreased BDNF protein levels and had no significant effect on AChE activity, compared to that in the vehicle-treated group.

**Conclusions:**

These results suggest that GO significantly improves novel object recognition as well as increases cell proliferation and neuroblast differentiation, by modulating hippocampal BDNF protein levels and AChE activity, while CO impairs novel object recognition and decreases cell proliferation and neuroblast differentiation, by reducing BDNF protein levels in the hippocampus.

## Background

In mice, neurogenesis is complete within 21 days after birth, yet adult brains retain their ability for neurogenesis in some specific areas, including the subgranular zone of the dentate gyrus and the subventricular zone of the lateral ventricle [1, 2]. These endogenous origins of neurogenesis seem to be an ideal source of compensatory repair and system functionality. For example, newborn cells in the subgranular zone of the dentate gyrus can migrate to the granular cell layer, where they integrate into the neuronal circuitry of the dentate gyrus as granule neurons [3]. These integrated neurons can then play a role in memory formation in the hippocampus. Adult neurogenesis can be affected by variables, such as environmental factors, growth factors, neurotransmitters, and external stimuli that alter the affective state of an animal [4–6]. Herbal supplementation can also modulate neurogenesis and thereby affect hippocampal functions, such as memory [7–9]. However, few studies have focused on the oil components of foods consumed in high quantities in oriental countries.

Vegetables of the genus *Allium*, such as garlic, onions, green onions, and chives, have been used as food additives in China and Egypt, and are now being studied for the prevention of infection [10–12] and cancer [13, 14]. The characteristic odor of garlic and chives arises from allicin (allyl 2-propene thiosulfinate or diallyl thiosulfinate) and other oil-soluble sulfur components, such as diallyl disulfide (DADS), diallyl trisulfide (DATS), and diallyl sulfide (DAS) [15–18]. These oil-soluble components exhibit antioxidant properties, by reducing the levels of reactive oxygen species and increasing glutathione-*S*-transferase expression [19, 20]. In previous studies, we demonstrated that *S*-allyl-L-cysteine, a water-soluble component of *Allium* species [21, 22], promotes cell proliferation and neuroblast differentiation in the dentate gyrus [7]. In addition, we have shown the neuroprotective effects of *Z*-ajoene, an oil-soluble component, against ischemic damage in the gerbil hippocampus [9]. On the other hand, the administration of 10 mg/kg DADS affects neurogenesis, by reducing hippocampal brain-derived neurotrophic factor (BDNF) levels, and impairs performance in the passive avoidance test [23]. In addition, the garlic essential oil (GO) compounds, DAS and DADS, promote the intrinsic calpain-caspase cascade for apoptosis in human neuroblastoma SH-SY5Y cells [24].

However, few studies have been conducted on the effects of oils from garlic or chives extracts on neurogenesis and their related mechanisms of action in the dentate gyrus. In the present study, we investigated the effects of GO and chives essential oil (CO) on cell proliferation and neuroblast differentiation in the naïve mouse, and considered possible mechanisms responsible for these effects in the dentate gyrus.

## Methods

### Experimental animals

Male C57BL/6 mice were purchased from Japan SLC Inc. (Shizuoka, Japan). They were housed under standard conditions with temperature (22 °C) and humidity (60 %) control, a 12-h light/dark cycle, and free access to food and water. The handling and care of the animals conformed to the guidelines complying with current international laws and policies (NIH Guide for the Care and Use of Laboratory Animals, NIH Publication No. 85–23, 1985, revised 1996) and were approved by the Institutional Animal Care and Use Committee (IACUC) of Seoul National University (Approval number: SNU-141112-1). During all of the experiments, every effort was made to minimize the number of animals used and the suffering caused by the procedures employed in the present study.

### Preparation of essential garlic and chives oils

Garlic (*Allium sativum*) and chives (*Allium hookeri*) (1 kg) were purchased from a local market in South Korea. They were authenticated by two oriental medicine doctors (Dr. Gwang Lim Choi and Cheol Soo Lee, Kyung-Dong Oriental Medical Clinic, Seoul South Korea) and the voucher specimen was deposited in our laboratory (deposition number: 2015–003). The oils can be obtained upon request via email to the corresponding authors. Both essential oils were obtained by steam distillation from *Allium sativum* and *Allium hookeri*. Briefly, the crushed garlic clove (200 g) or chives (200 g) was placed in a 2 L round-bottom flask and distilled water was added (400 mL) with boiling bubble stone. Then, the mixture was sonicated for 20 min. Finally, the mixture of garlic-water or chives-water was distilled for 4 h at 100 °C using a Clevenger-type apparatus, according to the method of Dadalioglu and Evrendilek [25].

### Treatment with essential garlic or chives oil

The animals were divided into 3 groups (*n* = 10 in each group): vehicle (corn oil)-, 10 ml/kg GO-, and 10 ml/kg CO-treated group. Vehicle, GO, or CO was administered orally to 9-week-old mice once a day for 3 weeks. The dosage was chosen, because oral administration of garlic oil (5 ml/kg body weight) daily for 3 months has been shown to significantly improve NaNO_2_-induced neurobiochemical disorders and oxidative stress [26]. The schedule was adopted, because doublecortin (DCX) is exclusively expressed in immature neurons from 1 to 28 days of cell age [27, 28].

### Novel object recognition test

The apparatus consisted of an acrylic box with three opaque walls and one transparent wall (45 × 45 × 30 cm). The floor was covered with woodchip bedding, which was mixed between trials and testing days to prevent the build-up of odor in any particular place. The test objects were made of solid metal and they could not be displaced by the mice because of their weight. The objects were cleaned with bleach to remove residual odors.

At 20 days of vehicle, GO, or CO treatment (1 h after dose was provided), each mouse (*n* = 10 per group) was allowed to explore the apparatus for 2 min. On the testing day (at 21 days of treatment), 1 h following the last dose, a session of two 2-min trials was performed. In the “sample” trial (T1), two identical objects were placed in two opposite corners of the apparatus. A mouse was placed in the apparatus and was left to explore these two identical objects. After T1, the mouse was placed back in its home cage for an inter-trial interval of 1 h. Subsequently, a “choice” trial (T2) was performed. In T2, a new object (N) replaced one of the objects presented in T1. The mice were exposed to two different objects: the familiar (F) and the new one (N). Exploration was defined as directing the nose towards the object at a distance of no more than 2 cm and/or touching the object with the nose. From this measure a series of variables were then calculated: the total time spent exploring the two identical objects in T1 and the time spent exploring the two different objects, F and N, in T2.

The preference for F and N in T2 was determined, by comparing the time spent exploring F with that spent exploring N. The discrimination index (DI) represents the difference in exploration time expressed as a proportion of the total time spent exploring the two objects in T2.

### Tissue processing

For histology, the animals (*n* = 5 in each group) from the vehicle-, GO-, or CO-treated groups were anesthetized with 1 g/kg urethane (Sigma-Aldrich, St. Louis, MO, USA) 2 h after novel object recognition test and perfused transcardially with 0.1 M of phosphate-buffered saline (PBS, pH 7.4) followed by 4 % paraformaldehyde in 0.1 M phosphate-buffer (pH 7.4). Brains were removed and post-fixed in a fixative for 12 h. Brain tissues were cryoprotected with 30 % sucrose overnight. Brain sections of 30-μm thickness were serially cut in the coronal plane using a cryostat (Leica, Wetzlar, Germany). Sections were collected in six-well plates containing PBS for further processing.

### Immunohistochemistry

In order to obtain accurate data for immunohistochemistry, free-floating sections were carefully processed under the same conditions. For each animal, tissue sections were selected between 1.46 mm and 2.46 mm posterior to bregma, by referring to the mouse atlas [29]. Ten sections, 90 μm apart from each other, were obtained and sequentially treated with 0.3 % hydrogen peroxide in 0.05 M PBS and 10 % horse serum in 0.05 M PBS. They were then incubated overnight with diluted rabbit anti-Ki67 antibody (1:1,000; Abcam, Cambridge, UK) or goat anti-DCX antibody (1:50; Santa Cruz Biotechnology, Santa Cruz, CA, USA), and subsequently exposed to biotinylated rabbit anti-goat or goat anti-rabbit IgG (diluted 1:200; Vector, Burlingame, CA, USA) and streptavidin peroxidase complex (diluted 1:200, Vector). Then, the sections were visualized by reaction with 3,3′-diaminobenzidine tetrahydrochloride (Sigma-Aldrich).

Number of Ki67- and DCX-positive cells was counted in each section of the dentate gyrus by using an image analysis system equipped with a computer-based CCD camera (software: Optimas 6.5, CyberMetrics, Scottsdale, AZ, USA). Cell counts from all of the sections of all of the mice in each group were averaged.

### BDNF protein levels in the hippocampal homogenates

For BDNF protein level and acetylcholinesterase (AChE) activity analyses, the animals (*n* = 5 in each group) from the vehicle-, GO-, or CO-treated groups were anesthetized with 2 g/kg urethane (Sigma-Aldrich) at 2 h after novel object recognition test and the hippocampi were dissected from the brain tissue. Left and right side of the hippocampus was used for BDNF protein levels and AChE activity assessment, respectively. BDNF protein level in the left part of the hippocampus was measured using a BDNF Emax immunoassay kit (Promega, Madison, WI, USA), as described previously [30]. Briefly, the tissue samples were weighed and 300 μL of lysis buffer was added to each sample. The samples were then sonicated for 30 s and centrifuged at 4 °C for 20 min. The supernatant was stored at −20 °C until it was analyzed. All samples were assayed in duplicate and the absorbance was read with an enzyme-linked immunosorbent assay (ELISA) plate reader (BioTek, Winooski, VT, USA). Total protein concentrations were estimated using BioRAD procedure (Hercules, CA, USA). The concentration of each sample was calculated by plotting the absorbance values on a standard curve generated by the assay.

### Measurement of AChE activity in the hippocampus

For measuring of the AChE activity in the synaptosome, the right part of hippocampus was homogenized in 10 volumes of an ice-cold medium, consisting of 320 mM sucrose, 0.1 mM EDTA, and 5 mM 4-(2-hydroxyethyl)-1-piperazineethanesulfonic acid (pH 7.5) in a motor driven Teflon-glass homogenizer. The synaptosomes were isolated, as described previously [31], using a discontinuous Percoll gradient. The pellet was suspended in an isoosmotic solution and the final protein concentration was adjusted to 0.5 mg/mL. Synaptosomes were prepared fresh daily, maintained at 4 °C throughout the procedure and used for enzymatic assays. The AChE enzymatic assay outcome was determined with a modified spectrophotometric method [32, 33].

The reaction mixture (2 mL final volume) contained 100 mM K^+^-phosphate buffer (pH 7.5) and 1 mM 5,5′-dithio-bis-(2-nitrobenzoic) acid. The catalytic activity was measured based on AChE reaction with 5,5′-dithio-bis-(2-nitrobenzoic acid) during 2-min incubation period at 25 °C, by the measurement of absorbance at 412 nm of the yellow anion, 2-nitro-5-thio-benzoate, produced from thiocholine. The enzyme was pre-incubated for 2 min. The reaction was initiated by adding 0.8 mM acetylthiocholine iodide (ATCh). All samples were run in triplicate and enzyme activity was expressed in μmol ATCh∙h^−1^/mg of protein.

### Statistical analysis

Data are expressed as means ± standard error of the mean (SEM). Differences among the means were statistically analyzed with a one-way analysis of variance, followed by Bonferroni’s post-hoc test, in order to compare the effects of GO and CO on: novel object recognition, cell proliferation, neuroblast differentiation, BDNF protein levels, and AChE activity in mice, using GraphPad Prism 5.01 software (GraphPad Software, Inc., La Jolla, CA, USA). The statistical significance level was set at *P* < 0.05.

## Results

### Effects of GO or CO on novel object recognition

**Fig. 1:**
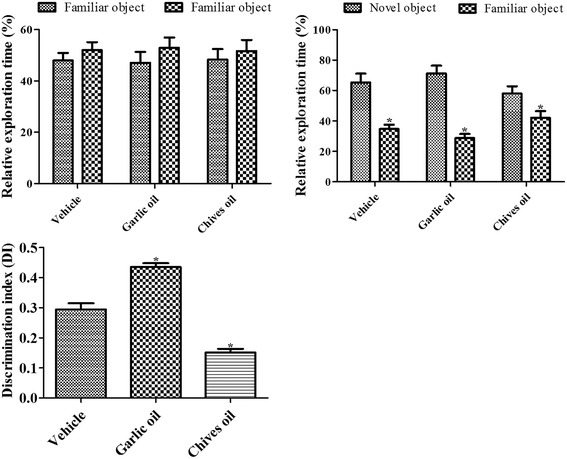
The effect of vehicle, garlic essential oil and chives essential oil on exploration time and discrimination index of familiar vs. novel objects in the novel object recognition test in mice (*n* = 10 per group; *p < 0.05 vs. familiar object on exploration time or vs. vehicle-treated group in discrimination index). Data for exploration time for each object (one object was replaced by a new one on the testing day) are presented as a percentage of total exploration time. All data are shown as mean exploration time ± SEM

During the training period, all mice from the vehicle-, GO-, or CO-treated groups spent the same amount of time exploring the two objects; however, during the test period, mice spent more time exploring the novel object than the familiar one. However, the proportions of time were different between the vehicle-, GO-, and CO-treated groups. The mice from the GO-treated group spent significantly more time exploring the novel object than mice in the vehicle-treated group. In contrast, mice in the CO-treated group spent significantly less time exploring the novel object than mice in the vehicle-treated group (Fig. 1). DI values significantly increased in the GO-treated group compared to that in the vehicle-treated group, while they significantly decreased in the CO-treated group (Fig. 1).

### Effects of GO or CO on cell proliferation

**Fig. 2:**
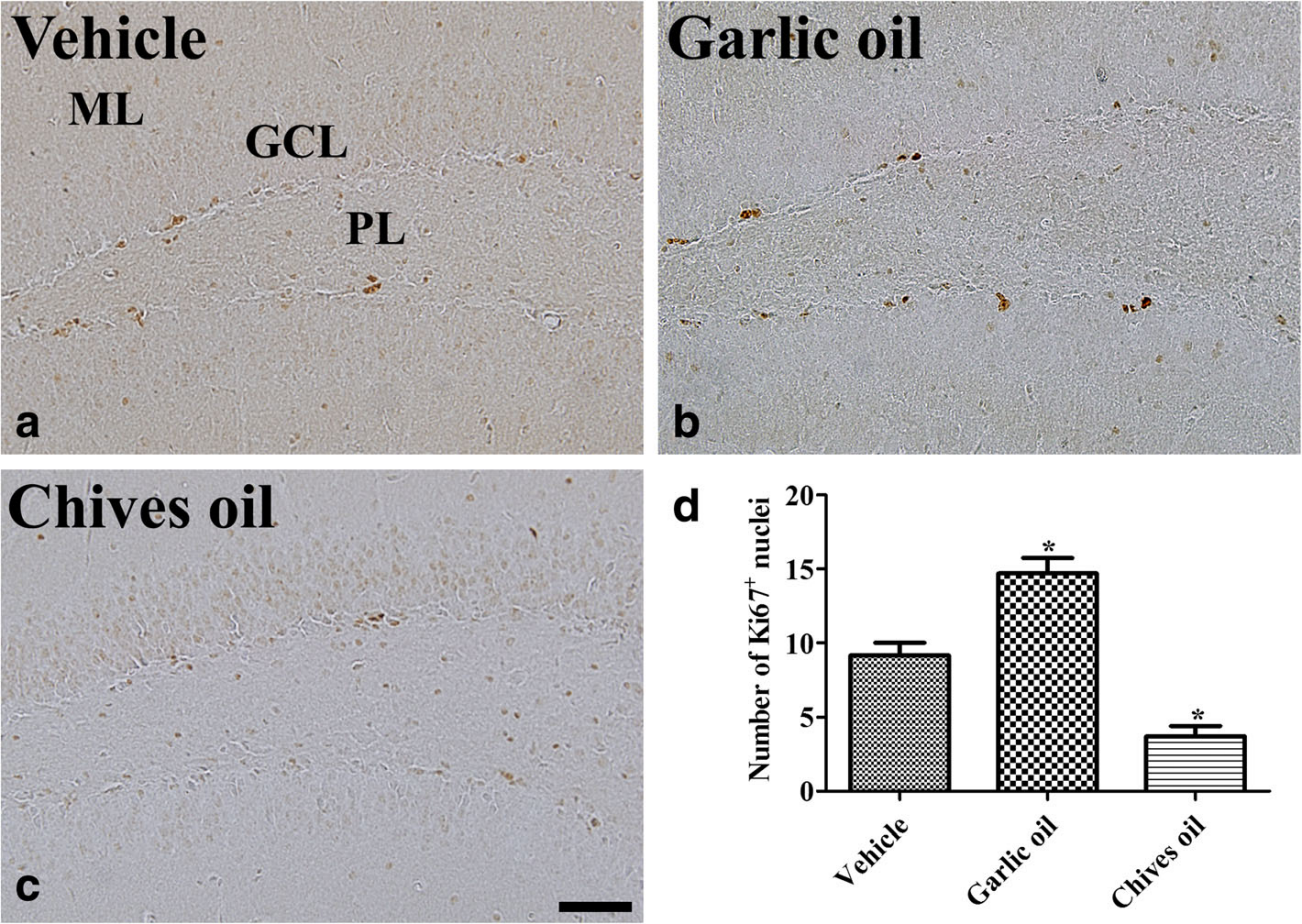
Immunohistochemistry for Ki67 in the dentate gyrus in the vehicle-treated (vehicle, **a**), garlic essential oil-treated (garlic oil, **b**) and chives essential oil-treated (chives oil, **c**) groups. Ki67-positive (^+^) nuclei are observed in the subgranular zone of dentate gyrus. Note that Ki67^+^ nuclei are abundant in the garlic essential oil-treated group, while in the chives essential oil-treated group, Ki67^+^ nuclei are few. GCL, granule cell layer; ML, molecular layer; PL, polymorphic layer. Scale bar = 50 μm. **d** Number of Ki67^+^ nuclei per section for each group (*n* = 5 per group; *p < 0.05, versus vehicle group). Data are presented as mean ± SEM

In the vehicle-treated group, Ki67-positive nuclei were found in the subgranular zone of the dentate gyrus (Fig. 2a) and the average number of Ki67-immunoreactive nuclei was 9.1 per section (Fig. 2d). In the subgranular zone of the dentate gyrus of the GO-treated group, Ki67-positive nuclei were more abundant in comparison to those in the in vehicle-treated group, and the average number of the nuclei was 14.7 per section (Fig. 2b and d). In the CO-treated group, few Ki67-positive nuclei were detected in the subgranular zone of the dentate gyrus and there were 3.8 positive nuclei per section (Fig. 2c and d).

### Effects of GO or CO on neuroblast differentiation

**Fig. 3:**
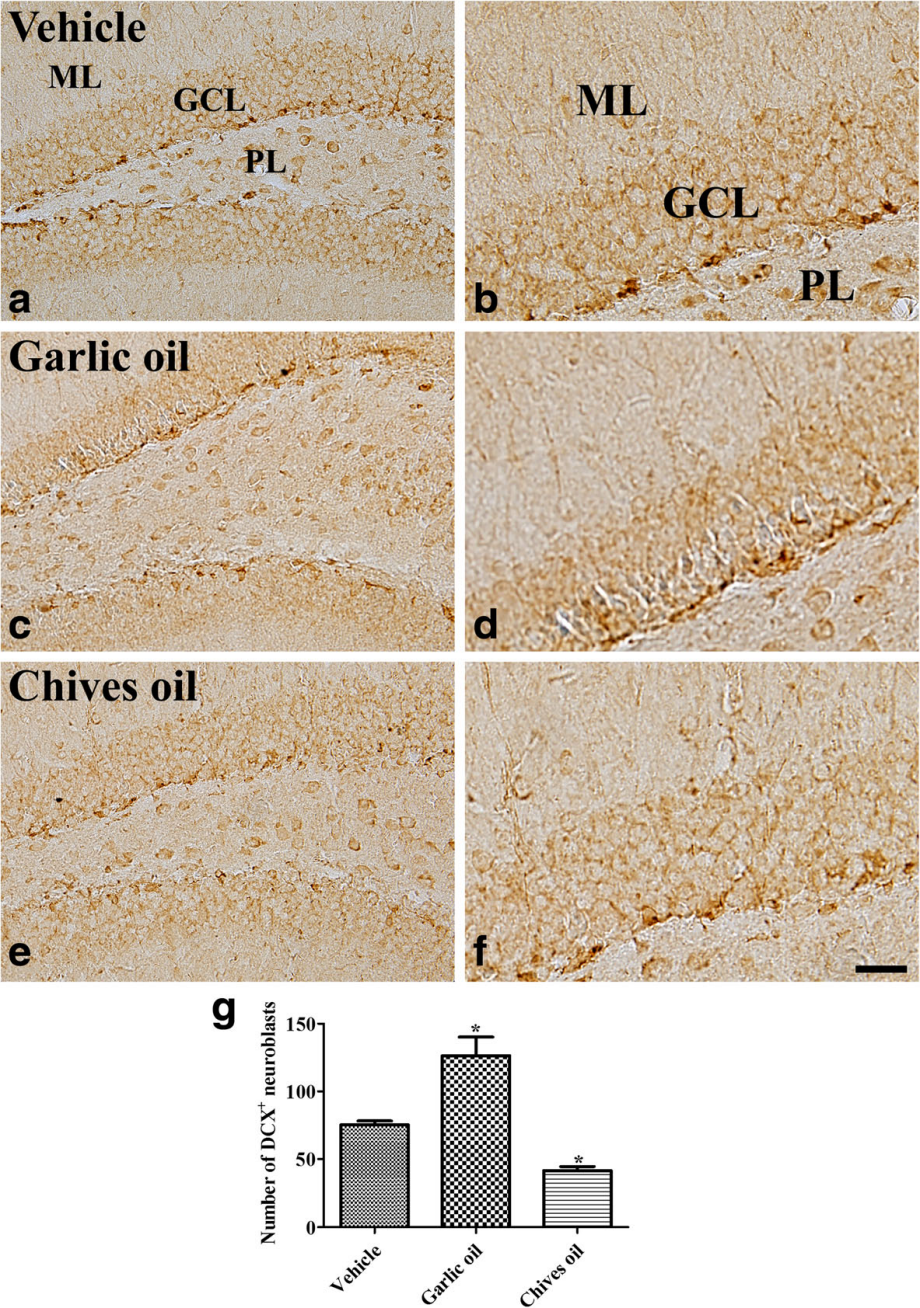
Immunohistochemistry for doublecortin (DCX) in the dentate gyrus in vehicle-treated (vehicle, **a**, and **b**), garlic essential oil-treated (garlic oil, **c**, and **d**) and chives essential oil-treated (chives oil, **e**, and **f**) groups. DCX-immunoreactive (^+^) neuroblasts are seen in the subgranular zone of the dentate gyrus and their dendrites are observed in the molecular layer (ML) of the dentate gyrus. Note that DCX^+^ neuroblasts and their dendrites are abundant in the garlic essential oil-treated group, while in the chives essential oil-treated group, DCX^+^ neuroblasts and their dendrites are few. GCL, granule cell layer; ML, molecular layer; PL, polymorphic layer. Scale bar = 50 μm (**a**, **c**, **e**), 25 μm (**b**, **d**, **f**). **g** Number of DCX^+^ neuroblasts per section for each group (*n* = 5 per group; *p < 0.05, versus vehicle group). Data are presented as mean ± SEM

In the vehicle-treated group, DCX-immunoreactive neuroblasts were detected in the subgranular zone of the dentate gyrus and their dendrites extended into the molecular layer of the dentate gyrus (Fig. 3a and b). In this group, the average number of DCX-immunoreactive neuroblasts was 75.4 per section (Fig. 3g). In the GO-treated group, DCX-immunoreactive neuroblasts were more abundant in the subgranular zone of the dentate gyrus compared to those of the vehicle-treated group (Fig. 3c and d) and the average number of DCX-immunoreactive neuroblasts was 126.4 per section (Fig. 3g). In the CO-treated group, only a few DCX-immunoreactive neuroblasts were detected in the subgranular zone of the dentate gyrus and their dendrites were poorly detected (Fig. 3e and f). In this group, the average number of DCX-immunoreactive neuroblasts was 41.5 per section (Fig. 3g).

### Effects of GO or CO on BDNF protein levels

**Fig. 4:**
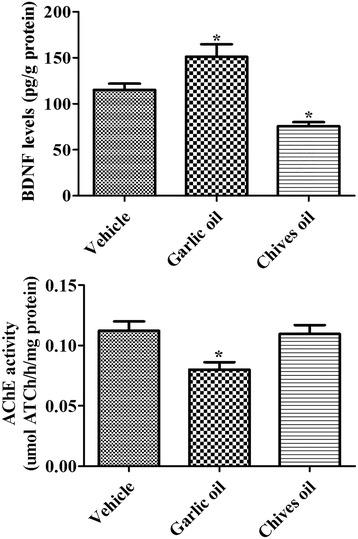
Brain-derived neurotrophic factor (BDNF) level and acetylcholinesterase (AChE) activity in synaptosomes of the hippocampi of vehicle-treated (vehicle), garlic essential oil-treated (garlic oil) and chives essential oil-treated (chives oil) groups (*n* = 5 per group; *p < 0.05, versus vehicle group). Data are presented as mean ± SEM

BDNF protein levels significantly increased in the dentate gyrus homogenates of the GO-treated group compared to those in the vehicle-treated group. In contrast, BDNF protein levels significantly decreased in the CO-treated group compared to those in the vehicle-treated group (Fig. 4).

### Effects of GO or CO on AChE activity

In the vehicle-treated group, AChE activity was 0.112 μmol ATCh∙h^−1^/mg protein in the hippocampal homogenates. In the GO-treated group, AChE activity in the synaptosome was significantly decreased, representing only 71.3 % of the activity of the vehicle-treated group. However, in the CO-treated group, AChE activity was similar to that in the vehicle-treated group (Fig. 4).

## Discussion

*Allium* species, such as garlic and chives, contain various organosulfur compounds, such as ajoene, vinyldithiins, DADS, and DATS [34–38]. In the present study, we focused on the effects of GO and CO on the hippocampus-dependent neurogenesis and memory formation in naïve mice. Significant improvements in the novel object recognition were observed in the GO-treated group, while the CO-treated group showed a significant reduction of the fraction of time spent exploring the novel object compared to that of the vehicle-treated group. These results are consistent with those of previous studies, which showed that sprouted or crude garlic extracts improved scopolamine-induced impairments of memory and cognition in mice [39]. It has also been reported that repeated administration of aged garlic extract enhanced memory function by increasing 5-hydroxytryptamine levels in rats [40]. Furthermore, commercial garlic extract powder capsules have been shown to improve cognitive function and brain mitochondrial function, which were impaired in obese, insulin-resistant rats because of high-fat diet [41].

The hippocampus is a major region related to memory formation and cognition, with continuous neurogenesis occurring in adult life [42–44]. It has been reported that enhanced hippocampal cell proliferation and neurogenesis improves memory deficits and memory impairments [45–48]. In this study, we observed significant increases in the number of cells positive for Ki67 and DCX, which are markers for proliferating cells and neuroblasts, respectively, in the subgranular zone of the dentate gyrus in the GO-treated group. However, in the CO-treated group, proliferation and differentiation significantly reduced compared to that of the control group. Previous studies have reported that garlic extracts and ascorbic acid ameliorated lead-induced neurotoxicity and decreased the number of DCX-positive neuroblasts [49]. These results suggest that increased cell proliferation and differentiation into DCX-positive neuroblasts by GO treatment may be related to the improvement of novel object recognition in the intact hippocampus. However, CO treatment may reduce novel object recognition in the hippocampus of naïve mice.

To identify possible mechanisms responsible for the enhancement of neurogenesis by GO in this study, we examined changes in the BDNF levels in the hippocampus. BDNF is implicated, as a potent neurotrophic factor regulating adult neurogenesis as well as synaptic transmission in the brain [50–55]. In addition, acetylcholine (ACh) and AChE activity are related to hippocampal neurogenesis [56–58]. Overexpression of the vesicular ACh transporter has been reported to enhance dendritic complexity in adult-born hippocampal neurons [59]. In addition, ACh enhanced cell proliferation and DCX-positive neuroblast production from neural stem cells *in vitro* [57, 60]. Furthermore, AChE inhibitors, such as donepezil hydrochloride, have been shown to enhance neurogenesis via downregulation of AChE activity in mice with vascular dementia [61, 62]. Finally, we have previously demonstrated that inhibition of AChE or butyrylcholinesterase activity significantly increases the cell proliferation and neuroblast differentiation in the dentate gyrus [63].

In the present study, we observed significantly increased BDNF levels in the hippocampus of the GO-treated group, but not in the CO-treated group, when compared to the control group. AChE activity was significantly decreased in the GO-treated group, but similarly detected in the CO-treated group, with the AChE activity inversely proportional to the BDNF levels in GO-treated group. Many previous studies have reported that BDNF may confer protective effects against various neurotoxic conditions in the brain, via reduction of the AChE levels or activity [64–66]. In addition, emerging evidence suggests that regulation of AChE activity by BDNF is related to hippocampal neurogenesis [61, 67–69]. For example, huperzine A, an AChE inhibitor, increases BDNF mRNA and protein levels, while cholinergic denervation or muscarinic antagonist treatment, such as atropine, decreases the hippocampal *BDNF* mRNA levels [70]. Interestingly, while garlic and chives are both representatives of the *Allium* species, their essential oils showed contrasting effects on novel object cognition, cell proliferation, neuroblast differentiation, BDNF levels, and AChE activity. In fact, it has been reported that various effects on neuroprotection, memory, and cell proliferation in the brain result from compounds of different *Allium* species. For example, *Z*-ajoene from garlic ameliorates the scopolamine-induced memory impairment dose-dependently [71]; however, alliin and DADS do not improve memory performance, cell proliferation, or neuroblast differentiation in the same model [23, 71, 72]. Therefore, the contradictory results of this study may be explained by the differing composition ratios and by how these compounds interact with memory formation in the naïve mouse. The exact mechanisms by which components induce the different effects of garlic and chives remain to be elucidated. In the present study, we chose one dosage of GO and CO. Test with varying doses would be needed for human trials, to use GO as functional food. In addition, the comparative chemical analytic studies of GO and CO will help detect possible toxic compounds in these *Allium* species.

## Conclusion

In conclusion, GO could be helpful to promote cell proliferation, neuroblast differentiation, novel object recognition, and memory formation by modulating BDNF levels and AChE activity in the hippocampus.

## Abbreviations

ACh: Acetylcholine
AChE: Acetylcholinesterase
ATCh: Acetylthiocholine iodide
BDNF: Brain-derived neurotrophic factor
DADS: Diallyl disulfide
DAS: Diallyl sulfide
DATS: Diallyl trisulfide
DCX: Doublecortin
SEM: Standard error of mean
